# Isoflurane anesthesia promotes cognitive impairment by inducing expression of β-amyloid protein-related factors in the hippocampus of aged rats

**DOI:** 10.1371/journal.pone.0175654

**Published:** 2017-04-12

**Authors:** Shuai Zhang, Xueyuan Hu, Wei Guan, Li Luan, Bei Li, Qichao Tang, Honggang Fan

**Affiliations:** Department of Veterinary Medicine, College of Veterinary Medicine, Northeast Agricultural University, Harbin, China; Universidade de Sao Paulo, BRAZIL

## Abstract

Isoflurane anesthesia has been shown to be responsible for cognitive impairment in Alzheimer’s disease (AD) and development of AD in the older age groups. However, the pathogenesis of AD-related cognitive impairments induced by isoflurane anesthesia remains elusive. Thus, this study was designed to investigate the mechanism by which isoflurane anesthesia caused AD-related cognitive impairments. Aged Wistar rats were randomly divided into 6 groups (n = 12), 1 control group (CONT) and 5 isoflurane treated (ISO) groups (ISO 0, ISO 0.5D, ISO 1D, ISO 3D and ISO 7D). The CONT group inhaled 30% O_2_ for 2 h without any anesthesia. ISO groups were placed under anesthesia with 3% isoflurane and then exposed to 1.5% isoflurane delivered in 30% O_2_ for 2 h. Rats in each ISO group were then analyzed immediately (ISO 0) or at various time points (0.5, 1, 3 or 7 day) after this exposure. Cognitive function was assessed using the Morris water maze test. Protein levels of amyloid precursor protein (APP), β-site APP cleavage enzyme-1 (BACE-1) and Aβ_42_ peptide were analyzed in hippocampal samples by Western blot. β-Amyloid (Abeta) plaques were detected in hippocampal sections by Congo red staining. Compared with controls, all ISO groups showed increased escape latency and impaired spatial memory. Isoflurane increased *APP* mRNA expression and APP protein depletion, promoting Aβ_42_ overproduction, oligomerization and accumulation. However, isoflurane did not affect BACE-1 expression. Abeta plaques were observed only in those ISO groups sacrificed at 3 or 7 d. Our data indicate that aged rats exposed to isoflurane had increased *APP* mRNA expression and APP protein depletion, with Aβ_42_ peptide overproduction and oligomerization, resulting in formation of Abeta plaques in the hippocampus. Such effects might have contributed to cognitive impairments, including in spatial memory, observed in these rats after isoflurane anesthesia.

## Introduction

Alzheimer’s disease (AD) is an irreversible neurodegenerative disease. It is primarily characterized by deleterious changes in orientation, judgment and personality, as well as social and cognitive abilities. Age is a major risk factor for AD, with AD being much more prevalent in older than in younger age groups. There is no United Nations (UN) standard numerical criterion, but the UN agreed cutoff is 60+ years to refer to the older population [1]. A current report estimated 5.3 million cases of AD in the United States with those over 65 years of age having about a 25 times greater incidence of the disease than those under 65 years of age [2].

The cellular etiology of AD is primarily related to loss of synapses and neurons in limbic and cortical structures, affecting the amygdala and hippocampus. A major molecular etiology of AD involves overproduction and accumulation of β-amyloid (Aβ) peptides. Aβ is formed primarily through serial proteolysis of the amyloid precursor protein (APP) by aspartyl protease β-site APP cleavage enzyme-1 (BACE-1) [3]. APP can be processed via two alternative pathways, the amyloidogenic pathway and the non-amyloidogenic pathway [4]. Under normal physiological conditions, APP was mainly through the non-amyloidogenic pathway and cleaved by α- and γ-secretases, which did not generate Aβ peptides. Under pathological conditions, APP was primarily by the amyloidogenic pathway and cleaved by β-secretase (BACE-1), which generated Aβ peptides. Extracellular Aβ exists in two major isoforms of the 42-residue Aβ_42_ and 40-residue Aβ_40_ [5]. Amyloid plaques consist primarily of Aβ_42_ and Aβ_40_, peptides generated by the amyloidogenic processing of APP [6]. Although levels of Aβ_40_ in the brain are higher than those of Aβ_42_, Aβ_42_ has greater neuronal toxicity because it more readily generates the large oligomers that form plaque-like deposits on the cell membrane surface [7–9]. In AD patients, BACE-1 expression in brain regions with Aβ deposition was 2–3 fold higher than in those regions without Aβ deposition [10]. BACE-1 is also a leading cause of Aβ overproduction and Aβ deposition in the brain which is a pathological hallmark of AD. Based on previous studies, the overproduction, oligomerization and accumulation of Aβ have become regarded as central to the pathogenesis of AD [11, 12].

Administration of general anesthesia is a risk factor for the development of AD. It is estimated that 200 million patients worldwide undergo general anesthesia for clinical surgery each year [13]. Studies on the relationship between anesthesia and AD also showed an increased incidence of AD in older patients undergoing general anesthesia, indicating a greater risk of general anesthesia in AD development [14, 15]. In addition, a previous study reported that patients undergoing coronary artery bypass graft surgery under general anesthesia were at increased risk for AD as compared to those under local anesthesia [16]. So, it is in urgent need to elucidate the mechanism by which general anesthesia caused cognitive impairment in the older patients, thereby contributing to exploring methods to reduce the risk factors for the development of AD.

Volatile anesthetics impaired spatial memory and increased cognitive impairment in aged rats [17, 18]. Isoflurane, a commonly used inhalational general anesthetic, caused hippocampal cell injury in adult rats, an effect that might have contributed to isoflurane-induced loss of cognitive function [19]. In another study, isoflurane induced increased Aβ expression, potentially promoting the development of neuropathogenesis [20]. However, the pathogenesis of anesthetic-induced cognitive impairment is not fully understood. There is no direct evidence linking β-amyloid protein, and its properties relevant to AD and isoflurane-induced cognitive impairment in aged rats. Thus, in this study, we investigated effects of isoflurane on APP, β-amyloid and BACE-1 expression and Abeta plaques formation in the aged rat hippocampus.

## Methods

### Animals

A total of 72 male Wistar rats, 24 months old and weighing 550 ± 50 g, were purchased from the Laboratory Animal Center of Harbin Medical University (Harbin, Heilongjiang, China). All rats were housed in standard polypropylene cages liberally lined with aspen wood shavings. They were maintained at 25°C under a 12 h/12 h light/dark cycle for at least 1 week before use in experiments. Food and water were available *ad libitum*. All work was approved by the Ethics Committee on the Care and Use of Animals, Northeast Agricultural University, Harbin, China.

### Experimental design

Aged Wistar rats were divided randomly into 6 groups (n = 12), 1 control group (CONT) and 5 isoflurane (ISO) groups. ISO groups were further divided into ISO 0, ISO 0.5D, ISO 1D, ISO 3D and ISO 7D groups, according to the day of sacrifice. To evaluate hippocampal-dependent spatial memory, 8 rats in each group were randomly selected for Morris water maze (MWM) training prior to test exposures. Thereafter, rats in the control group inhaled 30% O_2_ for 2 h, without any exposure to isoflurane anesthesia. And rats in ISO groups were placed under anesthesia with 3% isoflurane and then exposed to 1.5% isoflurane delivered in 30% O_2_ for 2 h. After isoflurane anesthesia, rats in ISO groups were maintained in 30% O_2_ for 20 min at 37°C to enable recovery from anesthesia. Finally, rats in the CONT group were sacrificed immediately after the control exposure and the cognitive testing. Rats in the ISO 0 group were sacrificed that day, immediately after the righting reflex recovery (time 0) and the cognitive testing, whereas rats in other ISO groups were given cognitive testing and sacrificed after 0.5 1, 3 or 7 day, respectively. After cognitive testing, all rats were euthanized at the indicated time and their hippocampi were harvested immediately. The right cerebral cortex and hippocampus of each brain were rapidly frozen in liquid nitrogen and stored at −80°C for protein analysis and quantitative real-time PCR analysis. The contralateral side was post-fixed in 4% paraformaldehyde saline and kept at 4°C for Abeta plaques analysis. Levels of *APP* and *BACE-1* mRNA in the hippocampal samples were measured by quantitative real-time PCR and those of APP, BACE-1 and Aβ protein by Western blot. Abeta plaques were visualized in hippocampal sections stained with Congo red.

### Isoflurane anesthesia

All rats were fasted, without water, for 12 h before isoflurane anesthesia or control exposure. Rats were placed in a temperature-controlled, sealed transparent anesthesia induction chamber with soda lime at the bottom. The side opening of this chamber was connected to an anesthesia machine. The rats were monitored physiologically during anesthesia, confirming that respiratory rate (RR), heart rate (HR) and pulse oximeter oxygen saturation (SpO_2_) remained in the safe range and rectal temperature was 37.0 ± 0.5°C. The minimum alveolar concentration (MAC) is the alveolar concentration at which 50% of animals do not show a motor response to painful stimuli. One MAC of isoflurane in rats is approximately 1.5% [21]. Rats in the CONT group inhaled only 30% O_2_ for 2 h at 37°C, whereas rats in ISO groups were placed under anesthesia with 3% isoflurane and then exposed to 1.5% isoflurane and 30% O_2_ for 2 h at 37°C. After isoflurane exposure, rats were maintained in 30% O_2_ for 20 min at 37°C to enable recovery from anesthesia.

### Morris water maze (MWM) experiments

The MWM test is widely used to assess spatial learning and memory in rodents [22]. To conduct this test, a circular pool (180-cm diameter, 50-cm depth) was filled with warm (26°C) opaque water. A hidden round platform (10-cm diameter) was submerged 2 cm below the surface of water, located in one quadrant. From each group, 8 rats were randomly selected for MWM training. Rats were trained using 4 trials per day for 5 consecutive days before receiving anesthesia or control exposure. The minimum interval between each trial was 15 min. For each trial, the rat was placed in 4 different quadrants of the water maze in turn and allowed to discover the hidden platform freely. Rats failing to discover the hidden platform within 90 sec were guided artificially to the hidden platform for 30 sec. On the sixth day, rats in ISO groups received isoflurane anesthesia or for the CONT group, the control exposure. The hidden platform was removed to perform the probe trial on day 0, 0.5, 1, 3 or 7 after isoflurane anesthesia and then rats were sacrificed. Swimming path, speed, latency (time to discover the hidden platform) and the number of platform crossings were recorded with a video tracking system (SuperMaze; Shanghai Xinruan Information Technology, Shanghai, China).

### Quantitative real-time PCR

Total RNA extraction and quantitative real-time PCR analysis were performed as previously described [23]. Total RNA was extracted from the hippocampus samples using the TransZol Up system (TransGen Biotech, Beijing, China). A GeneQuant 1300 spectrophotometer (GE Healthcare Bio-sciences AB, Uppsala, SE) was used to assess quantity and purity of the RNA. Special primers were designed and synthesized by Sangong Biotech (Shanghai, China) (Table 1). A 10-fold dilution series of the template was used during quantitative real-time PCR reactions to generate standard curves and the β-actin gene was used as an endogenous control. Transcripts were quantified using SYBR^®^ Premix DimerEraser^TM^ (TakaRa Biotechnology Inc., Dalian, China) on an ABI 7500 Real-time PCR System (Applied Biosystems). To quantify relative mRNA expression, the cycle threshold (CT) values of the target genes were normalized to the CT values of reference gene β-actin, and the results are presented as fold change using the 2^−ΔΔCT^ method. The relative mRNA expression of target gene in each group was calculated using the following equations: ΔCT = C_T target gene_ − C_T β-actin_, and ΔΔCT = ΔC_T treated group_ − ΔC_T control group_.

**Table 1:** Quantitative real-time PCR primers.

Gene	Primer (5′→3′)		Product size (bp)
β-actin	Forward	AGGGAAATCGTGCGTGACAT	163
	Reverse	CCTCGGGGCATCGGAA	
APP	Forward	GCAGAAGGACAGACAGCACA	140
	Reverse	GCAGGGACAGAGACTGGTTC	
BACE-1	Forward	AATCAGTCCTTCCGCATCAC	127
	Reverse	CTCCCATAACGGTGCCTGT	

### Western blotting

Western blot analysis was performed as previously described [24]. Hippocampal tissue samples were homogenized in an ice-cold RIPA buffer (Beyotime, Jiangsu, China) supplemented with a protease inhibitor. The homogenates were centrifuged at 12,000 × g for 5 min at 4°C, supernatants were collected and their protein concentrations determined with the BCA protein assay kit (Beyotime, Jiangsu, China). Proteins were separated by sodium dodecyl sulfate-polyacrylamide gel electrophoresis (SDS-PAGE), using 8% or 10% polyacrylamide gels, depending on the molecular weight separation range needed. After bands were transferred to polyvinylidene difluoride (PVDF) membranes, the membranes were incubated at 4°C for overnight with the appropriate primary antibody, anti-BACE-1 (CST, Danvers, USA), anti-APP (CST, Danvers, USA) or anti-Aβ_42_ (Abcam, Cambridge, UK), each diluted 1:500. To detect labeled protein bands, the membranes were next incubated for 2 h with the appropriate fluorescently labeled secondary antibody at room temperature. Image-Pro Plus 6.0 software **(**Media Cybernetics, Washington, USA) was used to analyze the fluorescence data for each blot. Anti-β-actin (1:750; ZSGB-BIO, Beijing, China) was also used as a protein loading control for each sample. Results are presented as the ratio of the intensity of the APP, BACE-1 and Aβ_42_ band to that of the β-actin band.

### Congo red staining for Abeta plaques

For Congo red staining, paraffin sections (5 μm) were treated as described [25]. Briefly, sections were deparaffinized in xylene and rehydrated. Sections were then treated with a working sodium chloride solution (sodium chloride-saturated 80% alcohol containing 0.01% sodium hydroxide) for 20 min at room temperature, stained for 1 h with 0.2% Congo red solution in NaCl-saturated 80% ethanol, and finally counterstained with hematoxylin, dehydrated in absolute alcohol. Images were captured using an Olympus BX41 microscope (Olympus, Tokyo, Japan) equipped with a Canon EOS 550D camera head (Canon, Tokyo, Japan) at a high-magnification field (400).

### Statistical analysis

SPSS Version 18.0 (Chicago, IL, USA) for Windows was used for statistical analysis. All data are presented as means ± standard deviation (SD). The escape latency and swimming speed during the training tests were averaged to give a mean value for testing on each day and these means were analyzed by one-way analysis of variance (ANOVA) with repeated measures. All other data were analyzed by one-way ANOVA followed by a Least Significant Difference post-hoc analysis without repeated measures. *P* values of <0.05 were considered to indicate significant differences.

## Results

### Isoflurane anesthesia induced spatial memory impairments in aged rats

**Fig 1:**
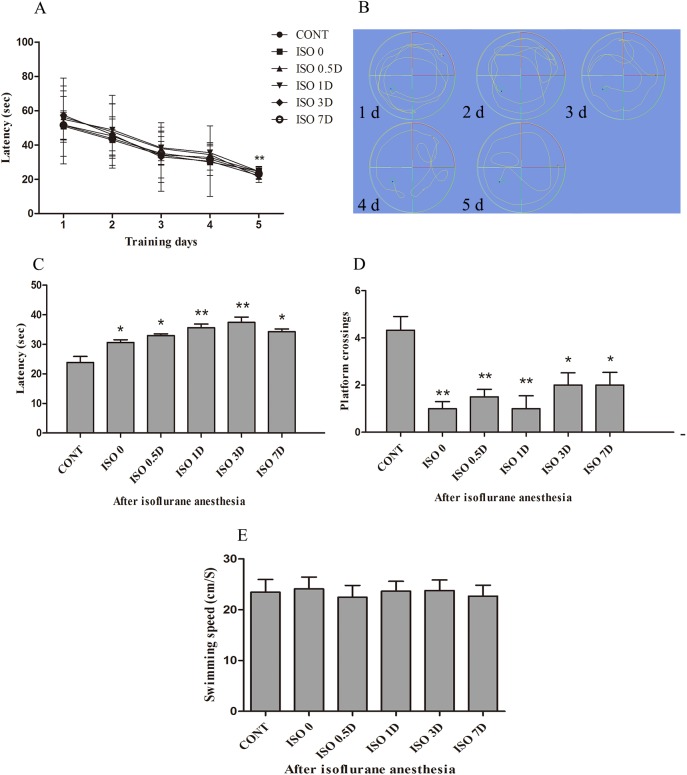
Isoflurane anesthesia induces the spatial memory impairment of aged rats. Spatial memory was examined by using the Morris water maze (MWM). (A) Latency to discover the hidden platform during training days. (B) Swimming paths during training days. (C) Latency to discover the hidden platform after isoflurane anesthesia. (D) The number of platform crossing in the probe trial tests. (E) Swimming speed during training days. Data are presented as the mean ± SD (n = 8). *P < 0.05, **P < 0.01 vs. the CONT group.

The MWM test is considered as a highly sensitive test of cognitive function and is widely used to elucidate hippocampus-dependent learning and memory in rodents. In our study, as shown in Fig 1A and 1B, the aged rats in each group, prior to anesthesia exposures, were able to discover the hidden platform after MWM training for 5 days. Compared with the latency on the first day, there was a significant decrease in latency on the fifth day in all rats (*P* < 0.01). On the fifth training day, aged rats in all 6 groups were able to discover the hidden platform within 30 sec. After isoflurane anesthesia, aged rats in the ISO 0, ISO 0.5D, ISO 1D, ISO 3D and ISO 7D groups had higher escape latency values than those in the CONT group (ISO 0, ISO 0.5D and ISO 7D, *P* < 0.05; ISO 1D, ISO 3D, *P* < 0.01) (Fig 1C). In the probe trial, the decreased number of platform crossings in the ISO 0, ISO 0.5D, ISO 1D, ISO 3D and ISO 7D groups were observed compared with the CONT group (ISO 3D, ISO 7D, *P* < 0.05; ISO 0, ISO 0.5D and ISO 1D, *P* < 0.01) (Fig 1D). Compared with the CONT group, there were no significant differences in the swimming speed of 5 ISO groups (ISO 0, ISO 0.5D, ISO 1D, ISO 3D and ISO 7D) (Fig 1E).

### Isoflurane anesthesia promoted the expression of *APP* but not *BACE-1* mRNA in the hippocampus of aged rats

**Fig 2:**
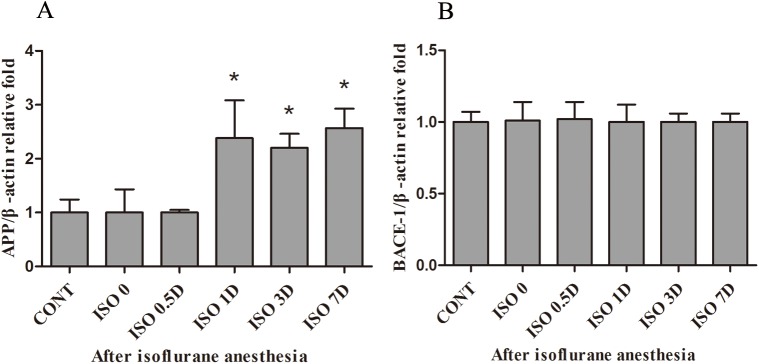
Effects of isoflurane anesthesia on the mRNA levels of *APP* and *BACE-1* in the hippocampus of aged rats. Twenty 4-month-old rats were exposed to 2% isoflurane anesthesia for 2 h. The expression of *APP* and *BACE-1* mRNA in the hippocampus of aged rats were measured by the quantitative real-time PCR. (A) The hippocampal *APP* mRNA expression increases significantly over time after isoflurane anesthesia. (B) There is no significant change in the level of *BACE-1* mRNA. Data are presented as the mean ± SD (n = 12 per group).

*APP* and *BACE-1* mRNA levels in the hippocampus were measured by quantitative real-time PCR (Fig 2). Compared with the CONT group, *APP* mRNA expression in the ISO 0 and ISO 0.5D groups was not changed, but it was significantly increased in the ISO 1D, ISO 3D and ISO 7D groups (*P* < 0.05) (Fig 2A). However, *APP* mRNA expression did not differ among the ISO 1D, ISO 3D and ISO 7D groups. There was no significant difference in *BACE-1* mRNA expression among all groups (Fig 2B).

### Isoflurane anesthesia promoted the depletion of APP protein and production of Aβ_42_ peptide, but not of BACE-1 protein, in the hippocampus of aged rats

**Fig 3:**
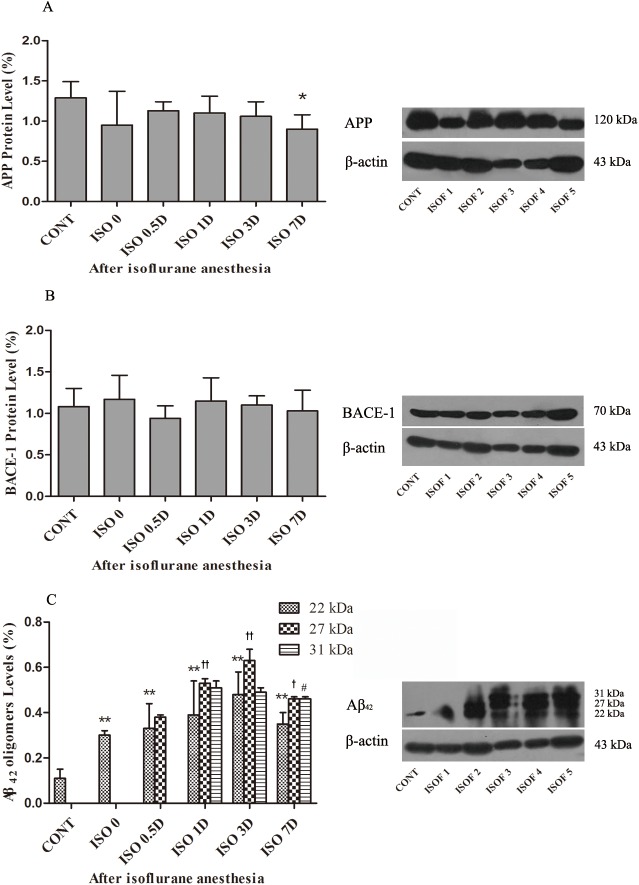
Effects of isoflurane anesthesia on the levels of APP, BACE-1 and Aβ_42_ oligomers protein in the hippocampus of aged rats. The levels of APP, BACE-1 and Aβ_42_ oligomers protein in the hippocampus of aged rats were measured by the Western blot. Representative results of APP, BACE-1 and Aβ_42_ oligomers protein expression in the hippocampus were shown (A), (B) and (C) respectively. Results are presented as the ratio of the intensity of the APP, BACE-1 and Aβ_42_ protein bands to the intensity of the β-actin protein bands respectively. Data are presented as the mean ± SD (n = 12 per group). *P < 0.05, **P < 0.01 vs. the CONT group. ^†^*P* < 0.05, ^††^*P* < 0.01 vs. the ISO 0.5D. ^#^*P* < 0.05, ^##^*P* < 0.01 vs. the ISO 1D.

APP, BACE-1 and Aβ_42_ levels in the hippocampus were measured by Western blot. Compared with the CONT group, the APP protein levels in all anesthesia-treated groups showed at least a trend toward a decrease, with a significant decrease in the ISO 7D group (*P* < 0.05) (Fig 3A). However, in agreement with the real-time PCR results, there were no significant differences in BACE-1 protein levels (Fig 3B). Aβ_42_ oligomers in the hippocampus were detected in various molecular weight bands ranging from 22 to 31 kDa (Fig 3C). The 22 kDa protein bands were present in all groups. Compared with the CONT group, the Aβ_42_ levels were significantly higher in the anesthesia-treated groups (ISO 0, ISO 0.5D, ISO 1D, ISO 3D and ISO 7D, *P* < 0.01) (Fig 3C) and were highest in the ISO 3D group. The 27 kDa protein bands existed in all except the CONT and ISO 0 groups. Aβ_42_ levels in other anesthesia-treated groups were significantly higher than in the ISO 0.5D group (ISO 1D, ISO 3D, *P* < 0.01; ISO 7D, *P* < 0.05) (Fig 3C). The 31 kDa protein bands were present in the ISO 1D, ISO 3D and ISO 7D groups. Compared with the ISO 1D group, Aβ_42_ levels were significantly lower in the ISO 3D and ISO 7D groups (*P* < 0.01) (Fig 3C). There is an upregulation in the overall levels of Aβ_42_ from the group ISO 0 to ISO 3D.

### Isoflurane anesthesia induced formation of Abeta plaques in the hippocampus of aged rats

**Fig 4:**
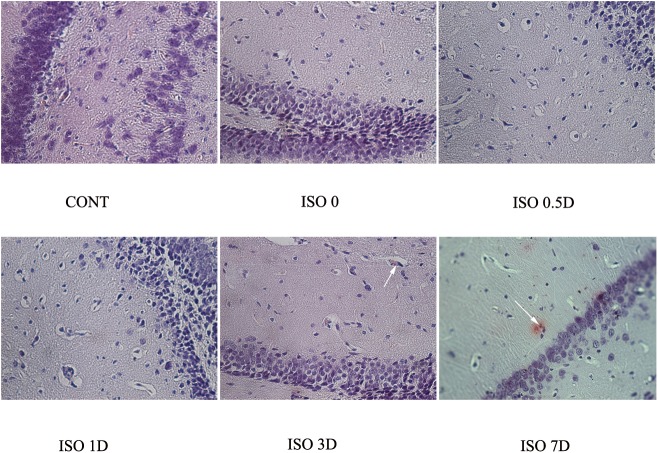
Localization of the Abeta plaques in hippocampus tissue slice after isoflurane anesthesia under an optical microscope (400). Detection of the Abeta plaques in the hippocampus of aged rats by Congo red staining. The Abeta plaques were staining orange red. Sections from the CONT, ISO 0, ISO 0.5D, ISO 1D, ISO 3D and ISO 7D were showed.

Paraffin-embedded 5-μm sections from the hippocampus were used to detect Abeta plaques, stained orange-red by Congo red staining (Fig 4). The Abeta plaques were visible under an optical microscope (400). Abeta plaques were observed in the hippocampi of rats in ISO 3D and ISO 7D groups. However, Abeta plaques were not observed in the CONT, ISO 0, ISO 0.5D or ISO 1D group.

## Discussion

In our study, we explored effects of isoflurane anesthesia on cognitive impairment in aged rats. We also examined the relationship between these cognitive impairments and certain AD-associated β-amyloid protein-related properties in the hippocampi of these animals.

Our study showed that isoflurane induced spatial memory impairments in the aged rats. The swimming speed was unaffected and maintained at a normal level after isoflurane anesthesia, indicating motor deficits in aged rats were not responsible for spatial memory impairments after test exposures. But longer escape latency and increased number of platform crossing in rats after isoflurane anesthesia were observed compared with the control group. Based on these results, we found that rats exposed to 1.5% isoflurane anesthesia for 2 h exhibited spatial memory impairments in the MWM test, indicating that proper dose of anesthesia can induce cognitive impairment in aged rats and this memory impairment in aged rats was mainly the disorder of evocation. Based on previous findings, it is evident that the commonly used inhaled anesthetic isoflurane can have an impact on the neuropathogenesis of AD [15, 21]. Although one previous study showed that isoflurane anesthesia for 4 h did not impair spatial reference learning of 2-month-old rats or affect long-term memory at 4 months [26], several other reports showed that isoflurane impaired learning and memory in rats [19, 21, 27, 28]. The discrepancies among these studies might be caused by differences in animal ages, methods, including the times at which tests were performed, and anesthesia concentrations. In addition, the escape latency in ISO 7D group and the number of platform crossings in ISO 3D, ISO 7D groups did not return to normal levels. From these results, we speculate that 1.5% isoflurane anesthesia for 2 h can induce transient cognitive impairments in aged rats. However, whether these effects keep more than one week is not yet clear.

APP may serve an essential role in the maintenance of synaptic function during ageing [29–31], but studies on APP mainly focused on its role in the pathogenesis of AD. Isoflurane was shown to promote abnormal APP processing and possibly to accelerate clinical progression of AD-related neurodegenerative disorders [14, 15, 32]. In our study, we found that 1.5% isoflurane anesthesia for 2 h increased *APP* mRNA but decreased APP protein expression (or increased APP depletion) in the hippocampus of aged rats. The correlation between mRNA and protein expression in multicellular organisms may not be consistent due to many factors including transcription efficiency, translation and degradation rates [33]. Moreover, APP protein was easy to be processed into downstream products via the amyloidogenic pathway or the non-amyloidogenic pathway [4]. In particular, we observed that APP protein expression in the isoflurane-treated groups showed a downward trend after anesthesia, but total amount of Aβ_42_ expression in the isoflurane-treated groups showed an upregulation after anesthesia. It was reported that exposure to 2% isoflurane for 6 h promoted APP processing and induced Aβ overproduction in H4 human neuroglioma cells stably transfected to express human wildtype full-length APP [15]. This is consistent with our finding that 1.5% isoflurane anesthesia for 2-h impacted APP processing in the aged rat hippocampus. Therefore, our data suggest that 1.5% isoflurane anesthesia increased APP depletion through the amyloidogenic pathway to induce Aβ_42_ overexpression in the aged rat hippocampus. It is notable that no significant effects of isoflurane on *BACE-1* mRNA or protein expression in the aged rat hippocampus were observed in our study. BACE expression is known to be elevated in the AD brain. A clinically relevant study indicated that isoflurane increased the levels of BACE and Aβ expression in the brain of C57/BL6 mice between 6 and 24 h following administration [20]. However, a follow-up study showed that isoflurane anesthesia did not affect BACE levels in the aged rat hippocampus [34]. Although the levels of BACE-1 were not changed in our study, we speculate that BACE-1 still catalyzed the APP progressing via the amyloidogenic pathway combined with the results of APP and Aβ_42_. In addition, isoflurane could induce β-Amyloid accumulation by enhancing levels of γ-secretase in H4 APP cells [35]. In our study, it is possibly that isoflurane alters the levels of γ**-**secretase and promote the APP progressing in the aged rat hippocampus.

Aβ, with a molecular weight of about 4.3 kDa, is a peptide with a folded configuration [36]. Aβ is primarily produced by serial proteolysis of APP by BACE-1. Monomeric Aβ in the brain can oligomerize into higher molecular weight forms, including dimers (8–10 kDa), trimers (13 kDa), tetramers (17 kDa), pentamers (22 kDa), hexamers (27 kDa), heptamers (31 kDa) and nonamers (40 kDa) [36, 37]. Emerging evidences accumulated in recent years demonstrated that amyloid beta-derived diffusible ligands (ADDLs, 15–56 kDa) have prominent neurotoxins in AD [36, 38, 39]. In our study, the primary Aβ_42_ oligomers detected in the hippocampi of aged rats after isoflurane anesthesia mainly were pentamers, hexamers and heptamers, belonging to ADDLs. ADDLs can cause mature neuronal damage, synaptic dysfunction and oxidative stress injury in the early of AD [36, 40, 41]. One previous report demonstrated that isoflurane enhanced Aβ oligomerization rates and increased its toxicity *in vitro* [42]. Another study also showed that repetitive 2% isoflurane exposure led to higher levels of Aβ oligomers in APP mice, compared with in wildtype mice [43]. Therefore, we propose that the overproduction of Aβ_42_ oligomers was caused by promotion of oligomerization of the peptide by isoflurane anesthesia. Furthermore, we observed increased levels of Aβ_42_ oligomers in the hippocampus after anesthesia at the same time that the aged rats exhibited significant cognitive impairments in the MWM test. Other studies also showed a close connection between increased Aβ oligomers and cognitive dysfunction [44, 45]. Endogenous Aβ_42_ oligomers were first positively identified in APP transgenic mice in year 2003 [46]. Early work revealed that, at high levels, Aβ_42_ induced an AD-like synaptic loss in transgenic mice without forming amyloid plaques [47]. Aβ_42_ oligomers derived from Tg2576 mice (APP-overexpressing transgenic mice) impaired memory or caused neuronal loss when administered to the brains of young wildtype rats, suggesting that these oligomers may have a causative effect on cognitive deficits associated with AD [48]. In more recent studies, soluble Aβ oligomers were implicated as synaptotoxins, potentially inducing an AD-related synapse failure and neurotoxicity, leading to cognitive impairment in AD [49]. The results of Congo red staining in our study were consistent with a role for Aβ oligomers in the neuropathology. We found significant Abeta plaques in the hippocampi of rats in the ISO 3D and ISO 7D groups, indicating formation of amyloid deposits by days 3 and 7. Thus, our results suggest that Aβ oligomers were important in isoflurane induced cognitive impairments in aged rats, indicating that these impairments may have an AD-related etiology.

In conclusion, in the aged rat hippocampus, isoflurane anesthesia increased the expression of *APP* mRNA and depletion of APP protein and promoted the overexpression and oligomerization of Aβ_42_ peptide, ultimately resulting in formation of Abeta plaques. These effects may contribute to the cognitive impairments observed in these rats after isoflurane anesthesia. Additionally, our findings should inform future investigations of the mechanism of isoflurane-induced cognitive impairment and help to elucidate the pathogenesis of AD in the older.
